# Olive leaf extract improves insulin sensitivity in overweight middle aged males; a randomized, double-blinded, placebo controlled, crossover trial

**DOI:** 10.1186/1687-9856-2013-S1-O34

**Published:** 2013-10-03

**Authors:** Martin de Bock, Jose Derraik, Christine Brennan, Eric Thorstensen, Wayne Cutfield

**Affiliations:** 1 Liggins Institute, University of Auckland, Auckland, New Zealand

## Aim

To investigate the impact of supplement olive leaf extract on insulin sensitivity in overweight middle aged males.

## Method

We conducted a randomized, double-blinded, placebo controlled, crossover trial on an overweight middle aged male population at risk of developing the metabolic syndrome comparing olive leaf extract containing 51.mg oleuropein and 9.7mg hydroxytyrosol to placebo. We measured insulin sensitivity (Matsuda method), pancreatic b-cell responsiveness (oral disposition index), lipid profile, ambulatory blood pressure, carotid intimal media thickness, body composition (DEXA) basal metabolic rate, hand-grip strength, wellness questionnaire, inflammatory cytokines, anti-oxidant potential all measured at baseline and end of both interventions.

## Results

38 participants were suitable for analysis. Even after controlling for total calorie intake fibre intake, physical activity, age, and percentage body fat (DEXA), insulin sensitivity was significant improved on treatment Vs placebo (19% p= <0.05), as well as the oral disposition index as a marker of pancreatic b-cell responsiveness (30% p=, 0.05). The cytokine profile was consistent with improved insulin sensitivity: acutely raised IL-6, IGFBP1 and IGFBP2. No other clinical outcomes were influenced.

## Conclusion

Olive leaf extract improves insulin sensitivity in overweight middle aged males. This is applicable to an overweight adolescent population.

